# What Is Required to End the AIDS Epidemic as a Public Health Threat by 2030? The Cost and Impact of the Fast-Track Approach

**DOI:** 10.1371/journal.pone.0154893

**Published:** 2016-05-09

**Authors:** John Stover, Lori Bollinger, Jose Antonio Izazola, Luiz Loures, Paul DeLay, Peter D. Ghys, Fast Track modeling working group

**Affiliations:** 1 Avenir Health, Glastonbury, Connecticut, United States of America; 2 United Nations Joint Programme on HIV/AIDS (UNAIDS), Geneva, Switzerland; 3 Independent consultant, Washington, DC, United States of America; British Columbia Centre for Excellence in HIV/AIDS, CANADA

## Abstract

In 2011 a new Investment Framework was proposed that described how the scale-up of key HIV interventions could dramatically reduce new HIV infections and AIDS-related deaths in low and middle income countries by 2015. This framework included ambitious coverage goals for prevention and treatment services for 2015, resulting in a reduction of new HIV infections by more than half, in line with the goals of the declaration of the UN High Level Meeting in June 2011. However, the approach suggested a leveling in the number of new infections at about 1 million annually—far from the UNAIDS goal of ending AIDS by 2030. In response, UNAIDS has developed the Fast-Track approach that is intended to provide a roadmap to the actions required to achieve this goal. The Fast-Track approach is predicated on a rapid scale-up of focused, effective prevention and treatment services over the next 5 years and then maintaining a high level of programme implementation until 2030. Fast-Track aims to reduce new infections and AIDS-related deaths by 90% from 2010 to 2030 and proposes a set of biomedical, behavioral and enabling intervention targets for 2020 and 2030 to achieve that goal, including the rapid scale-up initiative for antiretroviral treatment known as 90-90-90. Compared to a counterfactual scenario of constant coverage for all services at early-2015 levels, the Fast-Track approach would avert 18 million HIV infections and 11 million deaths from 2016 to 2030 globally. This paper describes the analysis that produced these targets and the estimated resources needed to achieve them in low- and middle-income countries. It indicates that it is possible to achieve these goals with a significant push to achieve rapid scale-up of key interventions between now and 2020. The annual resources required from all sources would rise to US$7.4Bn in low-income countries, US$8.2Bn in lower middle-income countries and US$10.5Bn in upper-middle-income-countries by 2020 before declining approximately 9% by 2030.

## Introduction

In 2011 a new Investment Framework for HIV/AIDS was proposed to guide efforts towards the rational use of resources to confront the AIDS epidemic [1]. The Investment Framework called for all low- and middle-income countries to focus on a set of Basic Programs of proven effectiveness; to implement social as well as program enablers, based on country-specific decisions about their implementation; and context-specific linkage of HIV interventions that support broader development objectives, such as social protection for children, reduction of gender-based violence, and health system strengthening.

The full implementation of the Investment Framework was expected to avert at least 12.2 million new infections and 7.4 million AIDS-related deaths by 2020, and thus provide a cost-effective means to achieve the goals of the 2011 United Nations General Assembly Political Declaration on HIV/AIDS [2] for 2015, such as reducing sexual transmission by 50%, reducing transmission among those who inject drugs by 50%, and virtual elimination of mother-to-child transmission according to the maximum expected availability of resources by 2015.

In 2014 an updated analysis was published which extended the time frame to 2050 and included new prevention approaches: Test and Treat, pre-exposure prophylaxis (PrEP) and potential AIDS vaccines [3]. That analysis examined the role of these new prevention technologies in getting to zero new infections. It concluded that new infections could be reduced to as low as 80,000 per year by 2050 with a more ambitious scale-up than the Investment Framework.

UNAIDS established a global goal of ending the AIDS epidemic as a public health threat by 2030, consistent with the three zeros vision: zero deaths, zero new infections and zero discrimination, operationalized as a 90% reduction of annual new HIV infections and AIDS-related deaths in 2030 compared to 2010. This paper examines what is needed in order to accomplish those goals with available technology and approaches (prophylactic or therapeutic vaccines are not included).

## Methods

We modeled future trends in annual new HIV infections and AIDS-related deaths using the Goals model, part of the Spectrum software package (version 5.41, available at www.AvenirHealth.org) [4], and applied an Excel version of the Resource Needs Model [5] to estimate the resources needed for program implementation. Goals is a simulation model that calculates HIV transmission among and between different population risk groups (monogamous heterosexual couples, those with multiple heterosexual partners, female sex workers and clients, men who have sex with men (MSM) and people who inject drugs (PWID)) on the basis of their behaviors (number of partners, contacts per partner, condom use, age at first sex, needle sharing) and characteristics that influence transmission (presence of other sexual transmitted infections, stage of infection, male circumcision, and use of antiretroviral therapy (ART) and PrEP). The effects of the bio-medical interventions are based mostly on results from randomized control trials that directly measured the effect on incidence. The effects of the behaviour change interventions are included both as direct effects on condom use and numbers of partners as well as part of the enabling environment that allows for greater uptake of interventions. We have included cash transfers for girls as several studies have shown reductions in incidence or changes towards safer behaviors. But because these effects seem to be limited to locations where school enrollment rates are low, we have assumed implementation only in countries with low rates of secondary enrollment for girls [6,7,8]. The coverage targets and the effects of the interventions are shown in Table 1. Complete details of the model are available elsewhere [3].

**Table 1:** Coverage goals and effects for the interventions included in this analysis.

Intervention	2020 Coverage	2030 Coverage	Effects
**KEY POPULATIONS**			
Service package for female sex workers	90%	90%	90% condom use at last sex act
Service package for MSM	90%	90%	90% condom use at last sex act
Service package for transgender populations	90%	90%	90% condom use at last sex act
Service package for PWID	90%	90%	90% condom use at last sex act, 51% reduction in percentage sharing needles
Opioid substitution therapy for PWID	40%	40%	46% reduction in number of sexual partners, 71% reduction in needle sharing
Service package for prisoners	90%	90%	Increased condom use in prisons
**BEHAVIOUR CHANGE INTERVENTIONS**			
Condom promotion	90% condom use at last sex	90% condom use at last sex	90% condom use at last sex among people with multiple partners
Cash transfers for girls	30% In Hyper-endemic countries with low rates of secondary school enrollment^1^	50% In Hyper-endemic countries with low rates of secondary school enrollment^1^	40% reduction in incidence among young women and girls (15–24 years old) in areas with low rates of secondary enrollment [6]
**MEDICAL INTERVENTIONS**			
PMTCT	95%	95%	80% starting ART before current pregnancy, 15% starting during current pregnancy. 98% reduction in perinatal transmission, 87% reduction in transmission during breastfeeding [9]
Male circumcision	90% of 10–29 year old men in countries with generalized epidemics and low MC rate^2^	90% of 10–29 year old men in countries with generalized epidemics and low MC rate^2^	60% reduction in susceptibility [10, 11, 12]
Post-exposure prophylaxis (PEP)	80%	80%	Provided to rape victims and health workers experiencing accidental exposure
PrEP for sero-discordant couples	10% in generalized and hyper-endemic countries	30% in generalized and hyper-endemic countries	80% reduction in susceptibility for sero-discordant couples. PrEP includes oral pills, vaginal gel, vaginal ring and injectable forms. [13, 14, 15, 16]
PrEP for sexually active females 15–24 in areas with incidence above 3% in this population group	10% in hyper-endemic countries	30% in hyper-endemic countries	80% reduction in susceptibility. For adolescent females we assume half this effect through 2020 then the full effect after 2020. PrEP includes oral pills, vaginal gel, vaginal ring and injectable forms. [13, 14, 15, 16]
Testing	24% of all adults and children in countries with generalized epidemics and of key populations and people with multiple partners in countries with concentrated epidemics	Gradual decrease to 20% of key populations, those with multiple partners and pregnant women in all countries with incidence below 0.1%. 20% of adults and children in countries with incidence above 0.1%	Identify HIV+ for linkage to care
Pre-ART care	81% of PLHIV not on ART	90% of PLHIV not on ART	
Adult ART	81% (90% started, 90% retained)	90% (95% started, 95% retained)	Eligibility for treatment expands to all PLHIV by 2018. 95% reduction in infectiousness among those virally suppressed [17]. By 2030 AIDS-specific mortality rates decline by 50% from 2015 rates due to enhanced retention and viral suppression.
**SOCIAL ENABLERS**	Includes community mobilization^3^, media communications^4^ and other general population approaches that support behavior change		

^1^ Hyper-endemic countries are: Botswana, Lesotho, Malawi, Mozambique, Namibia, South Africa, Swaziland, Zambia and Zimbabwe.

^2^ Countries include Botswana, Ethiopia (Gambela only), Kenya (Nyanza only), Lesotho, Malawi, Mozambique, Namibia, Rwanda, South Africa, South Sudan, Swaziland, United Republic of Tanzania, Uganda, Zambia and Zimbabwe.

^3^ Community mobilization can be divided into three categories: Outreach and peer communication and engagement activities; support activities; and advocacy, transparency and accountability. Community mobilization can be supported through community system strengthening which is a systematic approach to promote the development of informed, capable and coordinated communities and community based organizations. Hallmarks of effective community system strengthening include the involvement of a broad range of community actors and enabling them to contribute as equal partners alongside other actors to the long term sustainability of health and other interventions at community level. Community system strengthening aims to improve health outcomes by developing the role of key affected populations, communities and community based organizations in the design, delivery, monitoring and evaluation of services, activities and programs.

^4^ Media communication utilizes one or more channels to transmit a specific message to a large audience. Examples include brochures, billboards, posters, newspaper or magazine articles, comic books, television, radio, music videos, Internet, cell phones, songs, dramas, traditional and folk media, and interactive theatre. Media communication includes development of communication messages and materials and their transmission. Media communication seeks to promote positive changes in cognitive and behavioural outcomes such as increasing knowledge of modes of HIV transmission, increasing perceived risk of contracting HIV, reducing high-risk sexual behaviours such as having multiple partners, increasing positive protective behaviours such as condom use, and increasing the utilization of health care services. Media communication can also be utilized to create a supportive environment and often targets social, cultural and gender norms that may hinder behaviour change.

We applied the Goals model to 45 countries that together account for 86% of new infections globally:

- **East and Southern Africa**: Botswana, Ethiopia, Kenya, Lesotho, Malawi, Mozambique, Namibia, Rwanda, South Africa, Swaziland, United Republic of Tanzania, Uganda, Zambia, Zimbabwe
- **West and Central Africa**: Burkina Faso, Cameroon, Democratic Republic of the Congo, Ghana, Liberia, Nigeria, Sierra Leone
- **Latin America and the Caribbean**: Bolivia, Brazil, Costa Rica, Dominican Republic, El Salvador, Guatemala, Haiti, Jamaica, Mexico, Nicaragua, Panama
- **Asia and Pacific**: Bangladesh, Cambodia, China, India, Indonesia, Pakistan, Thailand, Viet Nam
- **Middle East and North Africa**: Egypt, Morocco
- **Eastern Europe**: Russian Federation, Ukraine
- **West and Central Europe and North America**: United States of America

The disease progression of the HIV+ population in the model is tracked by CD4 count. AIDS-related mortality is determined by CD4 count category and ART status and modeled as a competing risk with mortality from other causes. New child infections due to mother-to-child transmission are estimated based on the coverage of PMTCT services [9]. Data inputs for each country were drawn from national surveys and national progress reports (available at www.unaids.org) and adjusted to match the prevalence trends from national estimates as reported to UNAIDS. Country-specific coverage rates are based on country reports to UNAIDS and available at www.aidsinfoonline.org. When country-specific data were not available we used regional averages. Parameter values for progression and mortality rates are based on recommendations from the UNAIDS Reference Group on Estimates, Modeling and Projections [18] and are given in Tables A-I in Supporting Information File (S1 File). The impact results were adjusted for countries that were not explicitly modeled, so as to represent totals for 163 countries.

The resources required are estimated by intervention and year for each of the 117 low and middle- income countries (L&MICs). For most interventions the resources are estimated by multiplying the size of the target population (such as young women and girls, sex workers or men with multiple partners) by the coverage (the percentage reached with the intervention) and the unit cost (the cost to provide the service to one person for one year). Demographic data on the number of people by age and sex in each country are from the United Nations Population Division’s World Population Prospects 2015 [19]. Behavioral data and current coverage estimates are primarily from country reports as available in the UNAIDS on-line database ‘aidsinfoonline’.

General population interventions (community mobilization, voluntary medical male circumcision, HIV testing for general population, PrEP for serodiscordant couples) are to be implemented only in hyper-endemic and generalized epidemic countries. In generalized epidemics, incidence may vary substantially by geographic region. In those cases implementation efficiency was modeled to be improved by focusing the general population interventions on those areas of highest incidence. We have used survey data to estimate the proportion of the population living in the geographic areas accounting for at least two-thirds of people living with HIV. This percentage ranges from 41% and 43% in Kenya and Nigeria to 75% in the Côte d’Ivoire. For countries without data we used the average of those countries with data, 59%. Thus, in generalized epidemics the cost of these interventions is reduced by focusing the interventions in the areas that remain with high incidence. The interventions included, target coverage for all low- and middle-income countries and the effects are shown in Table 1. We also created a counter-factual scenario that assumes that coverage of all interventions remains constant at early-2015 levels through 2030.

Initial estimates of unit costs by intervention and country were based on a review of published and unpublished reports. An interactive database of unit costs is available at http://www.avenirhealth.org/PolicyTools/UC/. HIV Investment Cases conducted over the past 3 years provided updated information for 22 countries. The country-specific unit costs, current coverage levels and population size estimates were reviewed and revised as necessary by teams of national experts from 40 countries at regional meetings in Sub-Saharan Africa, Latin America and the Caribbean and Asia; a minority of countries did not request an update in the estimates. The regional average unit costs used in this analysis are shown in Tables 2 and 3.

**Table 2:** Regional average costs per person reached (in 2015 USD $).

Intervention	Asia and Pacific	East and Southern Africa	Eastern Europe & Central Asia	Latin America	Middle East North Africa	West and Central Africa	West and Central Europe and North America	Notes
**Key Populations**								
Service package for female sex workers	$ 77	$94	$108	$30	$15	$53	$180	1,5
Service package for MSM	$45	$101	$45	$44	$105	$51	$42	5
Service package for transgender populations	$45	$101	$45	$44	$105	$51	$42	5
Service package for PWID	$162	$135	$123	$49	$69	$90	$113	5
Opioid substitution therapy for PWID	$363	$265	$664	$664	$236	$265	$1,190	2
Service package for prisoners	$38	$31	$14	$10	$29	$1	$5	
**Biomedical interventions**								
PMTCT	$984	$365	$2,472	$1,967	$274	$585	$2,204	3
Voluntary medical male circumcision		$85						
Post-exposure prophylaxis	$100	$40	$101	$134	$137	$40	$101	
Pre-exposure prophylaxis	$200	$200	$200	$200	$200	$200	$200	
Condom promotion and supply	$0.12	$0.26	$0.27	$0.31	$0.29	$0.26	$0.27	
**Behavior change**								
Cash transfers for girls		$240						4
HIV testing services	$2	$9	$2	$2	$2	$8	$2	
**Social enablers**								
Community mobilization	$2	$4	$2	$6	$2	$2	$2	

All unit costs are expressed as the annual cost per person reached, except condoms are the cost per condom distributed.

Notes

1. Includes $5.92 for activities to prevent gender-based violence and assumes that 5% of condoms used are female condoms

2. We assume that 100% of costs are funded by AIDS budgets through 2020 declining to 30% by 2030.

3. The costs of PMTCT prophylaxis are shifted to the ART line item as Option B+ expands, leaving 10% of current cost by 2030 to cover the costs of syrup for the infants and nevirapine for those presenting too late to start ART.

4. 100% funded from AIDS budgets through 2020 declining to 30% by 2030

5. Unit costs decline by 2030 due to economies of scale by 16% for female sex workers, 19% for MSM and transgenders, 8% for PWID

**Table 3:** Current cost per patient per year for ART and pre-ART services. (2015 USD$).

Region	Service Delivery	Labs	ARVs—1st line	ARVs—2nd line	Pre-ART
Eastern Europe and Central Asia	$ 2,130	$ 255	$ 148	$ 684	$ 1,124
East Asia and Pacific	$ 109	$ 308	$ 136	$ 547	$ 444
Latin America and Caribbean	$ 1,725	$ 207	$ 634	$ 1,250	$ 910
Middle East and North Africa	$ 1,198	$ 144	$ 136	$ 328	$ 632
South and South-East Asia	$ 27	$ 140	$ 148	$ 400	$ 96
Sub-Saharan Africa	$ 222	$ 71	$ 136	$ 332	$ 182
Number of countries which contributed data	17	16	24	15	11

Notes:

These are costs in 2015 or latest year available.

Pre-ART costs largely disappear by 2020 as people would be started on ART as soon as they are identified as living with HIV; costs were kept constant.

In addition to the direct intervention costs we also include costs for Program Enablers, Social Enablers and Development Synergies. Program Enablers include planning and coordination, administration, supplies and logistics, staff training, M&E including surveillance and information systems. Social Enablers include communications for public awareness, advocacy and building political commitment, reform of laws and legal policies, stigma reduction and structural interventions. Development Synergies refers to activities wholly or partially supported by the AIDS budget that support broader development objectives such as support for orphans and vulnerable children, AIDS education, and prevention of violence against women. Costs for health system strengthening, while part of synergies, were included as a separate cost item. Cash transfers also have broader development objectives; as indicated in Table 2, financing for cash transfers drops from 100% funding under AIDS budgets during 2016–2020 to 30% by 2030.

Analysis and central reviews and revisions of country expenditure data collected through National AIDS Spending Assessments indicates that Program Enablers add 14% to direct intervention costs, Social Enablers add another 8% and Development Synergies add another 10.7%. We also included costs of health system strengthening rising from 6.1% of direct costs in 2015 to 9.7% by 2020 to support the rapid expansion required to reach these targets, and then declining back to 6.1% by 2030.

These projections assume that ART coverage continues the rapid scale up of the past few years so that the UNAIDS 90-90-90 treatment targets are achieved by 2020 at the same time as being consistent with WHO guidelines recommending therapy for all people living with HIV. [20] This means that 90% of all people living with HIV (PLHIV) are aware of their status, 90% of these are started and retained on ART treatment (together resulting in 81% ART coverage) and 90% of these achieve viral suppression by 2020. Once these targets are achieved we assume gradual improvements in service coverage and impact over the next ten year to 95-95-95 by 2030 (resulting in 90% ART coverage by 2030). In our projections all countries start at their current level of coverage and progress towards these targets by 2020 and 2030.

The effectiveness of ART in preventing transmission and HIV-related mortality is linked to the proportion of patients that achieve sustainable viral suppression. We assume that those patients who are virally suppressed do not transmit HIV [17]. The mortality of patients on ART is based on analysis of patient records by the IeDEA (International Epidemiologic Databases to Evaluate AIDS) Consortium [21]. Our model links viral suppression with both infectiousness and mortality. As a result current mortality rates are reduced by half by 2030 due to enhanced viral suppression.

Assumptions about the future costs of treatment were based on guidance from a committee of experts in ART costs (listed in Acknowledgements). The committee suggested that the cost of first line drugs in sub-Saharan Africa would decline by 55% to $75 by 2020 and that the cost of second line drugs would decline by 63% to $210 by 2020. For all other regions the same percentage decline is applied to current prices. The costs of third line drugs is assumed to be 10 times the cost of first line drugs. The future costs of service delivery and lab costs depends on the visit schedule. Many programs are making efforts to rationalize visit schedules to reduce the costs of ART. Based on WHO guidelines and the recommendations of our expert panel we assumed the following visit and lab schedule by 2020:

- Patients initiating ART: one CD4 test at initiation, 2 viral load tests (VL), four medical consultations, and two drug delivery/adherence support visits in the first year.
- Stable patients (assumed to be those virally suppressed): one VL test, 2 drug delivery/adherence support visits and 1.2 medical consultations per year.
- Patients not virally suppressed: 2 VL tests, 3 medical consultations and 2 drug delivery/adherence support visits per year
- HIV+ pregnant women: 6 medical consultations per pregnancy, one blood test, one test for early infant diagnosis.

We assumed future costs of $9.50 per CD4 test, $26 per viral load test and $20 per EID test. The costs per visit were calculated as 1/8 of the reported service delivery costs in 2015 (assuming an average of 8 visits per patient). We assume that by 2020, 30% of patient care will be delivered through community-based personnel at a cost 25% lower than facility-based care. Finally, we assumed that the percentage of patients on second line would increase to 13.5% by 2020 and the percentage on third line would increase to 3.4%. These assumptions result in the future costs of treatment shown in Table 4. The difficulties of finding and retaining patients to achieve 90-90-90 in 2020 are reflected in higher testing costs in 2020. After 2020 there may be higher marginal costs in 2030 but increasing efficiency of treatment for the existing patient population could still lead to small declines in average costs.

**Table 4:** Future costs of antiretroviral treatment, for different categories of patients, by region.

Region	New Patients	Stable Patients	Patients not Virally Suppressed	Pregnant Women (Incremental Costs)
Asia and Pacific	2020: $378	2020: $261	2020: $340	2020: $111
	2030: $341	2030: $224	2030: $340	2030: $111
Eastern Europe and Central Asia	2020: $1488	2020: $855	2020: $1268	2020: $627
	2030: $1440	2030: $806	2030: $1220	2030: $627
Latin America	2020: $1551	2020: $1059	2020: $1381	2020: $485
	2030: $1292	2030: $800	2030: $1220	2030: $485
Middle East and North Africa	2020: $1232	2020: $702	2020: $1049	2020: $524
	2030: $1206	2030: $524	2030: $1023	2030: $524
West and Central Africa	2020: $391	2020: $259	2020: $348	2020: $126
	2030: $379	2030: $247	2030: $336	2030: $126
East and Southern Africa	2020: $391	2020: $259	2020: $348	2020: $126
	2030: $379	2030: $247	2030: $336	2030: $126

## Results

**Fig 1:**
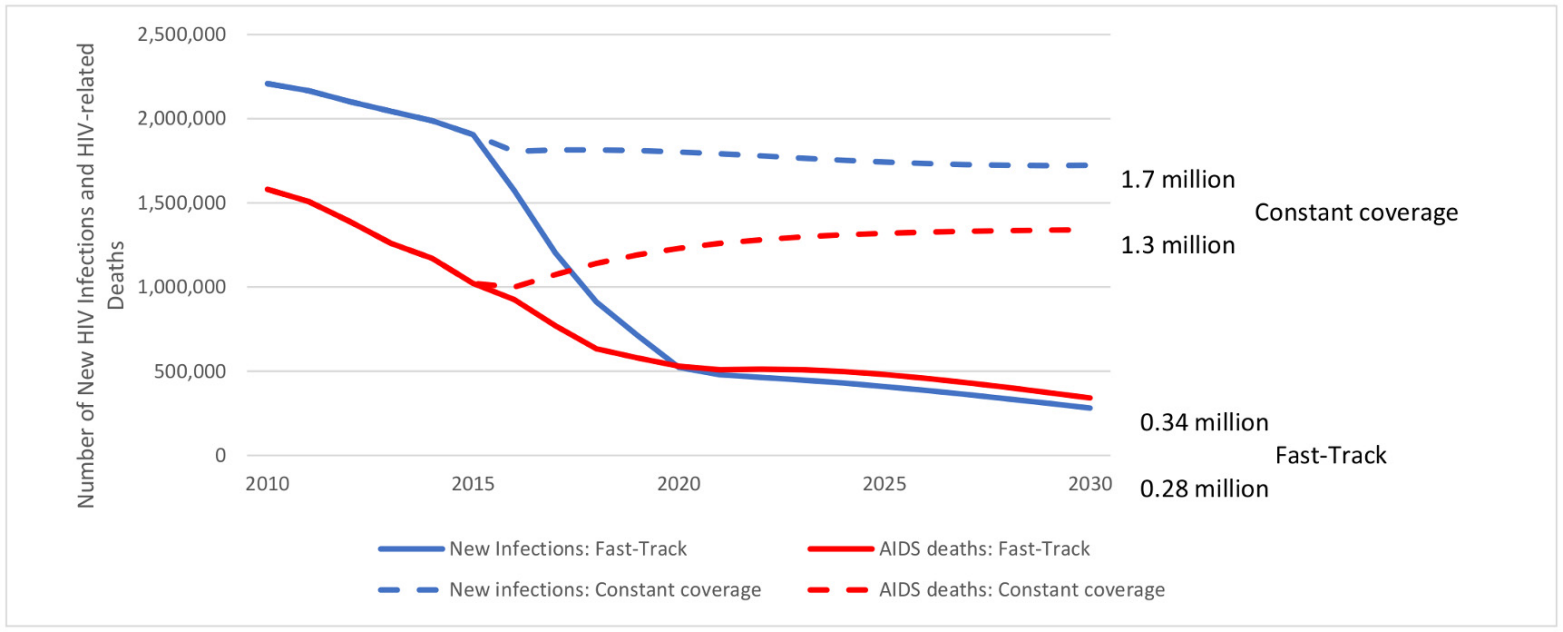
Trends in new HIV infections and AIDS-related deaths in low- and middle-income countries from 2010–2030, for the Fast-Track and constant coverage scenarios.

The scale up in prevention and treatment to the future levels described above would reduce new HIV infections in low- and middle-income countries from 2.2 million 2010 to about 280,000 by 2030, a reduction of nearly 90%. The number of new child infections decreases a bit more than the total, by 94%. The number of AIDS-related deaths would also be reduced sharply from 1.6 million in 2010 to 340,000 by 2030, or a reduction of nearly 80%, as shown in Fig 1. In both cases the sharpest reduction occurs by 2020 when most of the intervention scale-up targets are achieved. New infections continue to decline an additional 46% from 2020 to 2030 as a result of high coverage of prevention interventions and continued scale up of ART resulting in greater viral suppression. AIDS-related deaths stabilize after 2020 due to slower scale up of ART but eventually drop another 35% from 2020 to 2030. The percentage reductions are similar across all geographic regions so that by 2030 about 44% of new infections will be in East and Southern Africa, 12% in West and Central Africa and 16% in Asia and the Pacific.

Today about two-thirds of AIDS-related deaths occur to those who are not on ART. By 2030 there would be about an equal number of deaths among those few people who are not on ART and the much larger number receiving ART.

Fig 1 shows the trend in new HIV infections and AIDS-related deaths for both the Fast-Track and the counterfactual scenario. The constant coverage assumption in the counterfactual scenario leads to approximately constant incidence rates in most of the modeled countries reversing the downward trend of the past several years achieved by improving coverage rates. The adoption of the Fast-Track approach would result in averting11 million AIDS-related deaths and 18 million new HIV infections globally during the period 2016–2030; and 9.6 million and 15.1 million respectively in L&MICs.

In the countries in East and Southern Africa with high HIV prevalence and low levels of male circumcision, which account for about half of all new infections, under the Fast-Track approach 37% of infections averted are due to the use of effective ART, 37% due to condoms for those with multiple partners and 16% due to VMMC. The rest are averted by programs for key populations, PrEP and cash transfers. In generalized epidemics with already high levels of circumcision, 42% of infections averted are due to ART, 36% are due to condoms in multi-partner sex and 13% is due to programs for key populations. In concentrated epidemics, 48% of infections averted are due to ART, 26% to programs for key populations including PrEP and 18% to condoms for multi-partner sex.

Achieving these results will require significant increases in the number of people reached with prevention and treatment interventions. The service package for key populations is not limited to provision of condoms, safe needles or information and education, but also includes population services for discrimination reduction, promotion of access to testing, treatment and retention. Coverage of the service package for key populations (sex workers, men who have sex with men, transgender populations, people who inject drugs, prisoners) would increase by approximately 75% by 2030, while the number receiving opioid substitution therapy would rise by 160%. The population receiving cash transfers would rise to 5.5 million and the number receiving PrEP would rise to 13.5 million, mostly for discordant couples and key populations. The number of people receiving ART would rise to about 29 million by 2020 and remain at about that level by 2030. The number of people receiving ART would eventually decline as the result of fewer new infections, but that decline would occur well after 2030 when those started on treatment before 2020 reach old age and begin dying from other causes.

**Fig 2:**
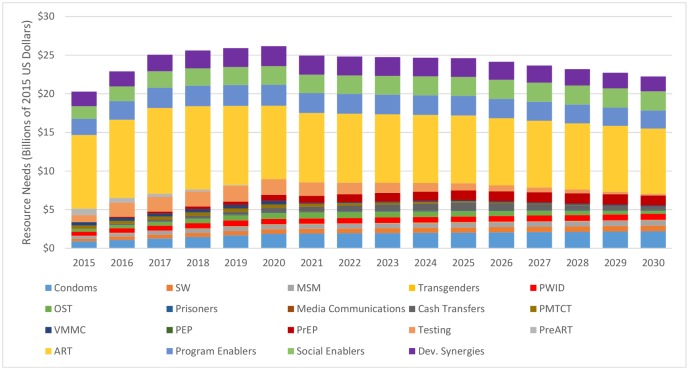
Annual Resource Needs by Intervention, 2013–2030. Key: SW = sex workers, MSM = men who have sex with men, PWID = people who inject drugs, OST = opioid substitution therapy, PMTCT = prevention of mother-to-child transmission, VMMC = voluntary medical male circumcision, PEP = post-exposure prophylaxis, PrEP = pre-exposure prophylaxis, Dev. Synergies = Development Synergies

The costs of achieving these increases in services in L&MICs are shown in Fig 2. These are estimates for all sources of financing including domestic government expenditures, donor assistance, private sector and out-of-pocket expenditures. The annual needs reach US$7.4 billion in Low-income countries, US$8.2 billion in lower middle-income countries and US$10.5 billion in upper-middle-income-countries by 2020 before declining approximately 9% in L&MICs by 2030. The decline is largely due to reductions in the unit costs of ART, reduced need for testing, a reduction in the proportion of opioid substitution therapy supported from the AIDS budget and the reduced need for pre-ART services and PMTCT due to the scale up of ART. Cumulative resource needs to 2030 are concentrated in a few interventions with two thirds needed for just 4 interventions: 39% for ART, 10% for program enablers, 9% for services for key populations and 7% for condoms. The resource needs for the 90/90/90 treatment components, including the basic programme components (pre-ART, ART and testing), the respective programme support costs, the appropriate share of the social enablers and shared costs of the outreach to key populations account for 78% in 2016, 67% in 2020 and 51% in 2030 in L&MICs.

**Fig 3:**
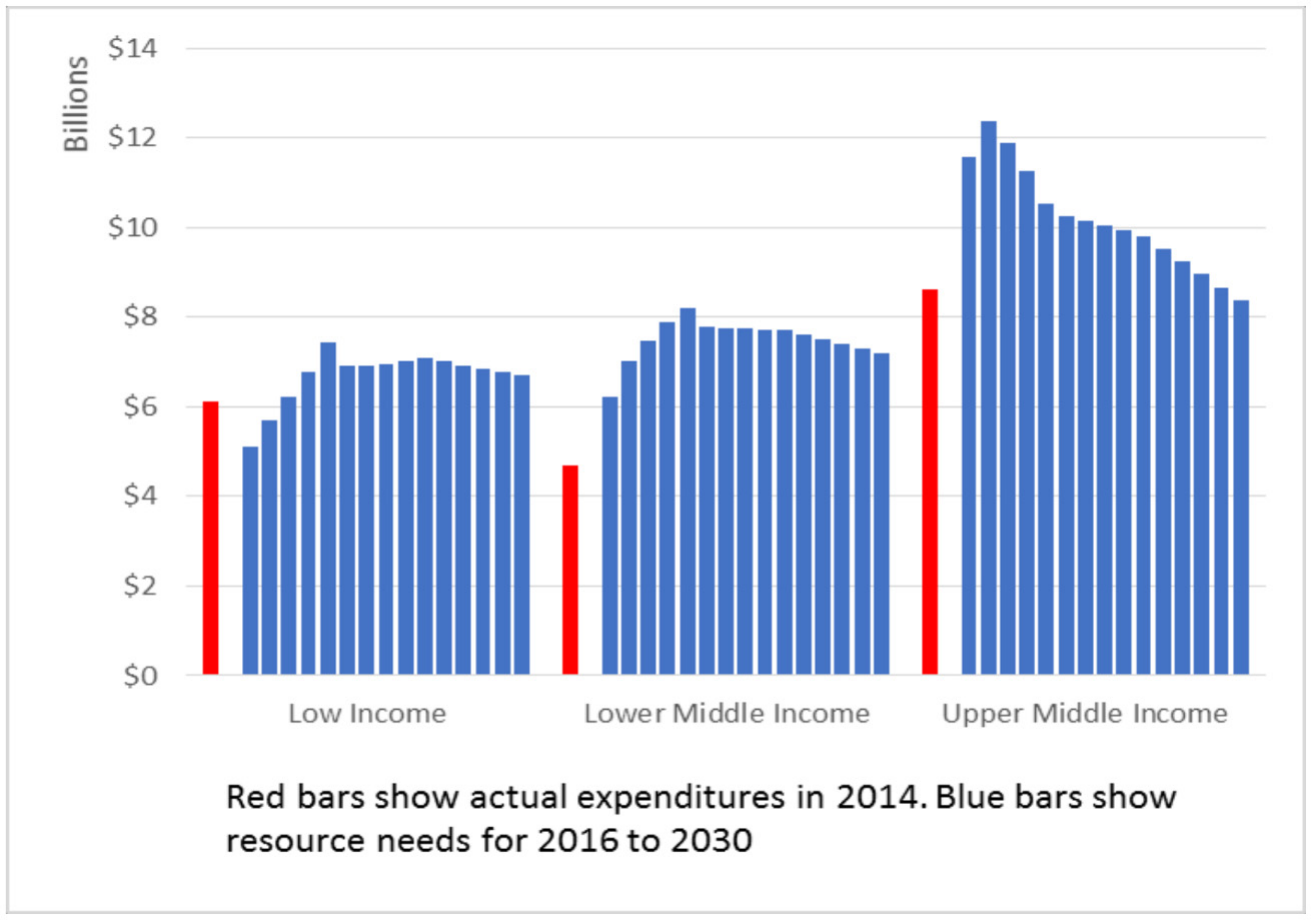
Resources available in 2014 and resources required from 2015–2030 by level of income in low- and middle-income countries (according to 2015 WB income level classification).

Resource needs are concentrated in East and Southern Africa (36%), Asia and the Pacific (21%), Latin America (17%) and West and Central Africa (16%). Over the entire time period, 27% of resources are needed in low income countries, 30% in lower middle income countries and 43% in upper middle income countries (Fig 3). Detailed tables showing the resource needs by country, intervention and year are available in Tables J-AE in the Supporting Information File (S1 File).

## Discussion

This analysis has shown what is required to achieve reductions in new infections and HIV-related deaths of 80–90% from 2010 to 2030. Achieving those goals will require rapid scale up to near universal coverage of key prevention and treatment interventions in all countries with large epidemics. Most important will be reaching targets for ART for all, voluntary medical male circumcision, PrEP, condoms, key populations and PMTCT.

Great progress has been achieved over the past decade. Continued rapid progress in the near future is dependent on multiple factors, especially increases in resources available for HIV programs and strengthened health systems to support service delivery.

However, keeping the coverage of the AIDS related services at the 2015 levels would imply that for many countries, the epidemic would increase. There would be 18 million more new infections and 11 million more AIDS deaths from 2010 to 2030 compared to achieving the Fast-Track targets globally.

One key factor for achieving a 90% reduction in new infections by 2030 is the need for a major effort to reach the target coverage levels between now and 2020. Rapid scale up of testing and treatment programs during this period will create the conditions to achieve the 90% reduction in new infections by 2030. A 10-year delay in reaching the 2020 targets would result in 3.2 million additional AIDS deaths and 6 million additional new infections in the 2016–2030 period.

Another key factor needed to generate the resources needed will be demonstrating that they are used effectively. We will need even greater efforts to improve cost-effectiveness by: i) targeting efforts to the geographic areas where they will have the greatest impact, ii) understanding the cost components of individual interventions and using that information to improve cost-effectiveness, iii) ensuring the right mix of interventions for the epidemic structure in each location, iv) continuing cooperation with manufacturers to ensure that prices for key ARVs and diagnostics are affordable to all, v) understanding costs at the facility level and above in order to optimize the mix of facilities providing key services, and vi) by adopting streamlined models of care without compromising the quality of services.

It should be noted that there are significant uncertainties in these projections. Although there is some uncertainty about the effectiveness of each of the interventions, the major source of uncertainty is whether programs can be scaled up as envisioned here. We did not include completely new interventions, such as prophylactic or therapeutic vaccines, that do not currently exist but could potentially make a contribution by 2030. We have limited the coverage of PrEP to 10% in 2020 and 30% in 2030 because of uncertainties about how quickly it can be implemented while addressing issues of adherence and costs. There is certainly the potential for future PrEP formulations with improved adherence and lower cost which could play a much larger role in achieving the desired reductions in incidence. Scaling up ART to the 90-90-90 targets by 2020 will require major efforts on the part of national programs, attention to human rights, expanded testing, improved adherence and retention, and a 50% increase in resources for treatment and testing. The significant contribution of voluntary medical male circumcision can only occur if programs become more effective at generating demand. Delays in achieving any of these targets will similarly delay the time by which these goals are achieved to well past 2030.

Today, two-thirds of all new infections occur in just 12 countries: South Africa, Nigeria, Russian Federation, Uganda, Mozambique, India, Indonesia, Zimbabwe, Kenya, Zambia, China and United Republic of Tanzania. Achieving these global impacts will require significant progress in each of these countries.

This analysis provides a roadmap for what is needed to end the AIDS epidemic as a global public health threat within the next 15 years as expressed in the Sustainable Development Goal 3.3 [22]. There are significant challenges ahead that must be addressed, but now we need to find the resources, the will, the human resources, the health and community systems and effective implementation approaches to make this a reality.

Current investments in the AIDS response are estimated at US$ 19.2 billion per year for the countries classified as low and middle-income countries in 2014, excluding the countries that have progressed to the high income category per the World Bank classification of countries. The resource needs for research and development are not included in these estimates. Compared to 2014 levels, an additional sum of US$6 billion in 2020 and US$ 2.9 billion in 2030 is needed. While these are large amounts, they are small compared to the amounts of public spending that countries devote to health.

In 2012, low- and middle-income countries spent more than USD$1.1 trillion on health from domestic resources. Projections of health expenditures per capita, regardless of the source, indicate that if the Fast-Track approach were fully funded, the HIV investment (adjusted to the health components) would be less than 4% of the total health expenditures in low income-countries, and less than 1% in lower-middle- and in upper-middle income- countries by 2020; by 2030 these percentages would decline to less than 1% in low-income countries, and less than a quarter of a percentage point in lower- and upper-middle-income countries respectively.

Providing the resource needs for critical enablers allows the full implementation of the critical basic programmes. For example, programme enablers include components that support the treatment programme, e.g. a functional supply chain of commodities (anti-retroviral medicine, testing kits, condoms, etc), quality assurance, training, and strategic information. Social programme enablers include community mobilization that includes the promotion of access to testing for key populations, as well as for young women and girls and broader population segments in countries with high HIV incidence or prevalence; in addition, community mobilisation includes support for adherence for ART services and for preventive services tailored according to the needs of specific population groups.

The frontloading of the resources needed to scale up the response by 2020 will be challenging. But this global effort will allow us to achieve the end of the AIDS epidemic as a global public health threat by 2030. There are few investments in global development that would have such a profound impact.

## Supporting Information

S1 File Supporting Information File with Tables SA-SAE with detailed inputs and outputs. (XLSX)
