# Correction: What Is Required to End the AIDS Epidemic as a Public Health Threat by 2030? The Cost and Impact of the Fast-Track Approach

**DOI:** 10.1371/journal.pone.0158253

**Published:** 2016-06-21

**Authors:** John Stover, Lori Bollinger, Jose Antonio Izazola, Luiz Loures, Paul DeLay, Peter D. Ghys, Fast Track modeling working group

There is an error in the S1 File from the list of Supporting Information. There is a formula error in the ‘Total by country’ tab which incorrectly links to the wrong rows. The corrected S1 File can be viewed below.

## Supporting Information

S1 File Supporting Information File with Tables SA-SAE with detailed inputs and outputs. This file includes supplementary data.
(XLSX)

## References

[1] Stover J, Bollinger L, Izazola JA, Loures L, DeLay P, Ghys PD, et al (2016) What Is Required to End the AIDS Epidemic as a Public Health Threat by 2030? The Cost and Impact of the Fast-Track Approach. PLoS ONE 11(5): e0154893 doi: 10.1371/journal.pone.0154893 27159260 10.1371/journal.pone.0154893 PMC4861332

