# Correction: What Is Required to End the AIDS Epidemic as a Public Health Threat by 2030? The Cost and Impact of the Fast-Track Approach

**DOI:** 10.1371/journal.pone.0213970

**Published:** 2019-03-14

**Authors:** John Stover, Lori Bollinger, Jose Antonio Izazola, Luiz Loures, Paul DeLay, Peter D. Ghys, Fast Track modeling working group

There is an error in Table 1. The sixth row under the “Medical Interventions” heading is missing. Please see the correct Table 1 here.

**Table 1:** Coverage goals and effects for the interventions included in this analysis.

Intervention	2020 Coverage	2030 Coverage	Effects
**KEY POPULATIONS**			
Service package for female sex workers	90%	90%	90% condom use at last sex act
Service package for MSM	90%	90%	90% condom use at last sex act
Service package for transgender populations	90%	90%	90% condom use at last sex act
Service package for PWID	90%	90%	90% condom use at last sex act, 51% reduction in percentage sharing needles
Opioid substitution therapy for PWID	40%	40%	46% reduction in number of sexual partners, 71% reduction in needle sharing
Service package for prisoners	90%	90%	Increased condom use in prisons
**BEHAVIOUR CHANGE INTERVENTIONS**			
Condom promotion	90% condom use at last sex	90% condom use at last sex	90% condom use at last sex among people with multiple partners
Cash transfers for girls	30% In Hyper-endemic countries with low rates of secondary school enrollment^1^	50% In Hyper-endemic countries with low rates of secondary school enrollment^1^	40% reduction in incidence among young women and girls (15–24 years old) in areas with low rates of secondary enrollment [6]
**MEDICAL INTERVENTIONS**			
PMTCT	95%	95%	80% starting ART before current pregnancy, 15% starting during current pregnancy. 98% reduction in perinatal transmission, 87% reduction in transmission during breastfeeding [9]
Male circumcision	90% of 10–29 year old men in countries with generalized epidemics and low MC rate^2^	90% of 10–29 year old men in countries with generalized epidemics and low MC rate^2^	60% reduction in susceptibility [10, 11, 12]
Post-exposure prophylaxis (PEP)	80%	80%	Provided to rape victims and health workers experiencing accidental exposure
PrEP for sero-discordant couples	10% in generalized and hyper-endemic countries	30% in generalized and hyper-endemic countries	80% reduction in susceptibility for sero-discordant couples. PrEP includes oral pills, vaginal gel, vaginal ring and injectable forms. [13, 14, 15, 16]
PrEP for sex workers, MSM, transgenders and PWID	10%	30%	80% reduction in susceptibility
PrEP for sexually active females 15–24 in areas with incidence above 3% in this population group	10% in hyper-endemic countries	30% in hyper-endemic countries	80% reduction in susceptibility. For adolescent females we assume half this effect through 2020 then the full effect after 2020. PrEP includes oral pills, vaginal gel, vaginal ring and injectable forms. [13, 14, 15, 16]
Testing	24% of all adults and children in countries with generalized epidemics and of key populations and people with multiple partners in countries with concentrated epidemics	Gradual decrease to 20% of key populations, those with multiple partners and pregnant women in all countries with incidence below 0.1%. 20% of adults and children in countries with incidence above 0.1%	Identify HIV+ for linkage to care
Pre-ART care	81% of PLHIV not on ART	90% of PLHIV not on ART	
Adult ART	81% (90% started, 90% retained)	90% (95% started, 95% retained)	Eligibility for treatment expands to all PLHIV by 2018. 95% reduction in infectiousness among those virally suppressed [17]. By 2030 AIDS-specific mortality rates decline by 50% from 2015 rates due to enhanced retention and viral suppression.
**SOCIAL ENABLERS**	Includes community mobilization^3^, media communications^4^ and other general population approaches that support behavior change		

^1^ Hyper-endemic countries are: Botswana, Lesotho, Malawi, Mozambique, Namibia, South Africa, Swaziland, Zambia and Zimbabwe.

^2^ Countries include Botswana, Ethiopia (Gambela only), Kenya (Nyanza only), Lesotho, Malawi, Mozambique, Namibia, Rwanda, South Africa, South Sudan, Swaziland, United Republic of Tanzania, Uganda, Zambia and Zimbabwe.

^3^ Community mobilization can be divided into three categories: Outreach and peer communication and engagement activities; support activities; and advocacy, transparency and accountability. Community mobilization can be supported through community system strengthening which is a systematic approach to promote the development of informed, capable and coordinated communities and community based organizations. Hallmarks of effective community system strengthening include the involvement of a broad range of community actors and enabling them to contribute as equal partners alongside other actors to the long term sustainability of health and other interventions at community level. Community system strengthening aims to improve health outcomes by developing the role of key affected populations, communities and community based organizations in the design, delivery, monitoring and evaluation of services, activities and programs.

^4^ Media communication utilizes one or more channels to transmit a specific message to a large audience. Examples include brochures, billboards, posters, newspaper or magazine articles, comic books, television, radio, music videos, Internet, cell phones, songs, dramas, traditional and folk media, and interactive theatre. Media communication includes development of communication messages and materials and their transmission. Media communication seeks to promote positive changes in cognitive and behavioural outcomes such as increasing knowledge of modes of HIV transmission, increasing perceived risk of contracting HIV, reducing high-risk sexual behaviours such as having multiple partners, increasing positive protective behaviours such as condom use, and increasing the utilization of health care services. Media communication can also be utilized to create a supportive environment and often targets social, cultural and gender norms that may hinder behaviour change.
